# Introductory Address

**Published:** 1859-01

**Authors:** B. G. Babington

**Affiliations:** President.


### TRANSACTIONS

OF THE

### EPIDEMIOLOGICAL SOCIETY OF LONDON.

---

### Papers and Communications.

---

### INTRODUCTORY ADDRESS AT THE OPENING OF THE SESSION 1858-9.

By B. G. BABINGTON, M.D., F.R.S., President.

[The opening meeting of this Society was held on Monday, Nov. 1st, at the room of the Royal Medical Benevolent College, Soho-square. After the usual preliminary business of the evening, Dr. Babington, the president, delivered the following address.]

Gentlemen,—When I had the honour of addressing you from this chair, in November last, I expressed a hope that the papers of the session, which we were then inaugurating, would not be less interesting and valuable than those of the session that had passed.

I feel I am justified in saying that my expectations have been fully realised; and that the papers read during the last session (the greater part of which have appeared in the *Sanitary Review*), have borne ample evidence of how much talent, industry and research, are still being devoted to the investigation of epidemiological questions.

On Monday, November 2nd, 1857, a paper "On the Study of Epidemic Diseases, illustrated by the Pestilences of London," was read by Dr. Greenhow.

On Monday, December 7th, 1857, Dr. Odling read a paper "On the District Mortality of London."

On Monday, January 4th, 1858, a paper "On the Causes which influence the Arrest or Spread of Cholera," by F. H. Johnson, Esq., of Bishopswearmouth, was read by Dr. McWilliam.

On Monday, February 1st, a paper "On the Influence of Drainage and Water Supply on Public Health" was read by Dr. Snow. And here, gentlemen, I cannot, in justice to my feelings, omit to notice the great loss which the public at large, as well as the profession, and in particular this society, have sustained in the early and lamented death of Dr. Snow. The sound knowledge of his profession which he possessed—his indefatigable industry, his unwearied zeal in the cause of medical science, demanded our admiration, while the liberality and the straightforward candour and honesty of purpose which marked his character, endeared him to a numerous circle of friends, and called forth our respect for his worth, even though we might occasionally differ from his views. Let us hope that his good deeds will avail him in the eyes of a Merciful Judge, and that he may meet the reward of the just in a better world.

On Monday, March 1st, Dr. Richardson read a paper "On the Investigation of Epidemic Diseases by Experiment."

On Monday, April 5th, a paper "On the Sickness and Mortality in the French Army in the East" was read by Dr. Milroy.

On Monday, May 3d, a paper "On Diphtherite, or Prevalent Sore-throat," was read by Dr. Camps.

On Monday, June 7th, a paper "On the Distribution of the Mortality from Hydrophobia in England, as an illustration of certain peculiarities in the mode of extension and prevalence of Epidemic Diseases; with suggestions for the better observation of Epidemics," was read by J. N. Radcliffe, Esq.

On Monday, July 5th, a paper "On the Difficulties attending the Study of Epidemic Diseases," by W. H. Michael, Esq., Mayor of Swansea, was read by Dr. McWilliam.

You are all well aware of the amount of time and labour which the society has devoted to the important subject of small-pox and vaccination; and I am sure you will be gratified to know, that just previous to the cession of office by Lord Palmerston's government, a deputation from the Small-pox and Vaccination Committee waited upon the Right Hon. William Cowper, then President of the General Board of Health, who promised the deputation, that, although he was about to leave office, his aid in Parliament should still be given towards the promotion of Vaccination. Since the accession to power of the present ministry, the Earl of Derby has received a deputation from the Society on the same question, and assured the deputation that the object they had in view should meet with the attention of Government.

Small-pox has been of late unusually prevalent in this country, more especially in Wales. From the Cape of Good Hope we also learn that Cape town, the capital of the colony, has been invaded by small-pox, and that the disease was spreading to an alarming extent in the interior districts. Government had taken measures to mitigate the ravages of the disease; but the want of vaccine lymph was much complained of. "Compulsion as regards vaccination," adds the reporter, "is lawful and just. Unvaccinated children are as dangerous as gunpowder to a neighbourhood." Diphtheria and scarlatina have prevailed and continue to prevail extensively in various parts of the country. Small-pox is also raging to an alarming extent at Jacmel, St. Domingo (Haiti).

I took occasion, at the opening meeting of the past session, while placing before you the leading events of the Society during the previous year, to draw your attention to the importance of noting the course of those epidemics which in that time had appeared in various quarters of the globe.

Among other epidemic visitations, I alluded to a fever of a very malignant character which was then devastating Lisbon. The disease, which I believe was true "yellow fever," broke out about the 9th of September, 1857, and the city was not declared healthy until the 24th of December following. We gather from the official reports, which most probably underrate the real state of the case, that during the 105 intervening days, there had occurred 13,482 cases of fever, of which 4759 had proved fatal. The weather had been for some time before the disease ceased, "cold, clear, and bracing."

The West India Islands, more especially the Island of St. Thomas, have been less unhealthy during the past year than for some previous years. Dr. Kinnear, of Jamaica Hospital, writes to Dr. McWilliam, under date October 11, 1858:—

"We have been very healthy here during this summer; and with exception of 76 cases of remittent fever from the *Tartar* and *Leopard* men of war, we have had little to do since the affair of the *Susquehanna*, in April last"; and Dr. Lawson at the same time informs Dr. McWilliam: "We continue remarkably healthy in Jamaica, I am happy to say, although there has been a moderate amount of disease among the black troops. There have been occasional fever cases among the officers, but as yet we have not lost a case since April; in fact, the disease has been particularly mild and manageable, or to speak more accurately, perhaps, has terminated favourably." The *Susquehanna* American frigate was, however, grievously attacked by this scourge while at Belize, in the early part of the year. The medical officers of the ship were prostrated by the disease; and after a large number of the crew had been treated at Port Royal Hospital, Jamaica, the ship proceeded to New York, in medical charge of Mr. Rose, assistant-surgeon of the Royal Navy.

It will be interesting to know, that in a communication from Dr. Kinnear, deputy inspector of the Royal Naval Hospital, received this day by our Secretary, he says: "We made no discoveries as regards treatment in the cases of the *Susquehanna.* I tried strychnine in several cases, and there was complete recovery in one, whilst it failed in others. There was no evidence of contagion, as the fever did not spread here, nor did it leave any trace behind. The vessel having been cleared out at New York, she was commissioned a few months after, and strange to say, she had forty cases of fever immediately, which proves that although clean and pleasant to the eye, she was foul in the bilges, *which were never opened up,* and the officers complained of the smell from them."

Dr. Lawson, Deputy Inspector-General of Hospitals and P.M.O. of the Station, thus writes from Jamaica to Dr. McWilliam, Oct. 9, 1858: "There will be a certain amount of obscurity about the fever in the *Susquehanna* which may never be completely cleared up,* and the explanation of the cause may consequently be doubtful." He adds—

"I have been working away with the microscope, and have hit on several things which I do not see mentioned elsewhere. One of these is, that every specimen of urine I have examined since July (and I did not make a special examination of the point before), has had a very considerable quantity of *hippuric acid* in it, generally with a good deal of uric acid and urea. The cases examined include black and white men labouring under fever, and a few cases of healthy people. In all the quantity was very decided, and was easily detected by placing a drop of urine on a piece of glass, adding to it half as much hydrochloric acid, and allowing it to evaporate spontaneously, or by employing heat for that purpose. The urea

* This refers to an alleged intercourse between the *Susquehanna* and the crew of one of the English mail packets, in which there was fever. (J. O. M'W.)

and other salts crystallise, and the hippuric acid is seen running through the mass in a form not unlike plumes of ostrich feathers. It requires a power of from 50 to 100 or more to familiarise the eye to the appearance; but when that is done, there is no difficulty in ascertaining its presence with a pocket lens, from the peculiar manner in which the whole crystallises."

Yellow fever also broke out some time in August last at Ferrol and Corunna, and in some of the villages of the seaboard of Gallicia, in Spain. Little or no information regarding this outbreak has as yet been obtained; but at the suggestion of the Spanish Consul in London (who, having himself been the subject of yellow fever during the terrible epidemic at Cadiz in 1800, takes great interest in the disease), our Secretary has addressed a letter on the subject to Dr. Mateo Seoane, of Madrid, *proto-medico* of the kingdom.

Yellow fever has been known to prevail at Santander and Guipuzcoa, on the north coast of Spain; but the great yellow fever fields in that country have been the provinces of Andalusia, Murcia, Valentia, Catalonia and Arragon, which run into each other, and are situated on the southern and eastern parts of the kingdom. So far as I am aware, there is no account of yellow fever having ever before existed in Gallicia.

The occurrence, in the course of the last summer, of yellow fever on the flats of Vera Cruz, in Mexico, and more recently at New Orleans,* surprised no one; but the experience of the last ten years has shown how this disorder can travel beyond its usual haunts.

Thus, in 1849, yellow fever appeared in Brazil, a country in which the disease had been unknown for at least 120 years. Some years later, the same pestilence found its way to the western side of the South American Continent. The disease still prevails in Brazil, and as I have already said, Lisbon was last year invaded by it. Newcastle, in Jamaica, the elevation of which (3800 feet) above the sea level had always before bestowed immunity from fever to its inhabitants, has twice within the last few years suffered from yellow fever.†

These, and other facts which might be mentioned, appear

---

* By late accounts, the yellow fever at New Orleans shews no sign of abatement. October 11th. Deaths from yellow fever yesterday, 55; for the week, 390. October 12th. The deaths by yellow fever in this city for the last thirty hours were 58.

† In the Appendix to Dr. Milroy's Report on the Cholera in Jamaica, occurs the following notice of some isolated cases of bad fever at Newcastle:—"If any other proof was wanted to shew that mere elevation of site will not suffice to maintain the health of troops in such a climate, the unexpected occurrence to me suggestive of the desirability of inquiring whether the views generally entertained with reference to the limitation of yellow fever, by elevation, temperature, latitude, soil, etc., do not admit of some modification. Above all, it seems necessary, with reference to an important question of the day, *Quarantine,* to ascertain, as far as possible, the manner by which this disorder reached those countries which it has recently visited either for the first time or after a long absence.

There have been some outbreaks, but nowhere to an epidemic extent, of cholera among our troops in India. Cholera has also prevailed at St. Petersburgh.

In the month of May last, a disease at first described as a kind of typhus fever, broke out at Bengazi, on the coast of Barbary. From the report of the commission despatched from Constantinople to investigate this epidemic, which proved to be true plague, it seems that the disease first appeared among a tribe of Bedouin Arabs, some miles from Bengazi, the town of which has a population of 10,000 souls, and stands on the seaboard of a low, vast plain. In six or eight days the disease had reached Bengazi; and before the end of May, the daily number of fatal cases had amounted to 29, which was, however, the highest number attained in one day during the whole course of the epidemy. By the middle of July, the average daily mortality had fallen to eight. Up to that period, the total mortality, so far as could be ascertained in the absence of regular registration, was 806. By a similarly approximate method of reckoning, the number attacked was 1340, which would raise the average deaths to 60 per cent. of attacks. As it is calculated that two-thirds of the whole population fled to Malta, Alexandria and elsewhere, it would result that 40 per cent. of those who remained were attacked. Many also took refuge in the interior of the country, and thus the contagion of the disorder was carried to Gharb, Derna, and other parts of the province. By the latter end of August, the disease had disappeared.

Such then, gentlemen, is a brief and necessarily imperfect outline of the main events of the Society during the session that has passed. The details connected with committees, donations, and financial and other considerations, belong more appropriately to the Council Report, and I have, therefore, not considered it necessary to do more than allude to them.

---

of several fatal cases of fever, with all the characters of yellow fever, in 1849, may well give rise to serious apprehensions for the future. These cases were clearly attributable to the operation of local impurities in the building where they occurred."

To those who have attended the meetings, I need make no reference to the excellence of the papers, which on those occasions have been submitted to them; but the sketch, however slight of the epidemics, which during the last twelve months have appeared in different parts of the world, may serve to remind us of the ample scope for epidemiological investigation, which still lies before us.

Before I conclude, I would fain be allowed to express a hope, that the present session may be characterised, not only by diligent and careful labour on the part of committees, but also by a more constant and regular attendance by the members at the ordinary meetings of the society.

We shall in vain look for adequate support from the profession and the public, if we ourselves do not bear in mind, that a proper and respectable assemblage of members at the meetings, is one of those elements in a society, upon which its life and prosperity largely depend.

